# Fabrication of biodegradable hollow microsphere composites made of polybutylene adipate co-terephthalate/polyvinylpyrrolidone for drug delivery and sustained release

**DOI:** 10.1016/j.mtbio.2023.100628

**Published:** 2023-04-06

**Authors:** Chuan Xie, Qinqin Xiong, Yuanzhi Wei, Xin Li, Jiajun Hu, Min He, Shinan Wei, Jia Yu, Sha Cheng, Mashaal Ahmad, Yufei Liu, Sihai Luo, Xi Zeng, Jie Yu, Heng Luo

**Affiliations:** a State Key Laboratory of Functions and Applications of Medicinal Plants, Guizhou Medical University, Guiyang, 550014, China; b Key Laboratory of Chemistry for Natural Products of Guizhou Province and Chinese Academy of Sciences, Guiyang, 550014, China; c Key Laboratory of Macrocyclic and Supramolecular Chemistry of Guizhou Province, Guizhou University, Guiyang, 550025, China; d Department of Polymer Material and Engineering, College of Materials and Metallurgy, Guizhou University, Guiyang, 550025, China; e Department of Chemistry, Norwegian University of Science and Technology (NTNU), 7491, Trondheim, Norway; f National Engineering Research Center for Compounding and Modification of Polymeric Materials, Guiyang, 550014, China

**Keywords:** Polybutylene adipate co-terephthalate (PBAT), Polyvinylpyrrolidone, Sustained-release drug carrier, Microspheres, Biodegradable

## Abstract

Sustained drug release has attracted increasing interest in targeted drug therapy. However, existing methods of drug therapy suffer drug action time, large fluctuations in the effective concentration of the drug, and the risk of side effects. Here, a biodegradable composite of polybutylene adipate co-terephthalate/polyvinylpyrrolidone (PBAT/PVP) consisting of electrospun hollow microspheres as sustained-released drug carriers is presented. The as-prepared PBAT/PVP composites show faster degradation rate and drug (Erlotinib) release than that of PBAT. Furthermore, PBAT/PVP composites loaded with Erlotinib provide sustained release effect, thus achieving a better efficacy than that after the direct injection of erlotinib due to the fact that the composites allow a high drug concentration in the tumor for a longer period. Hence, this work provides a potential effective solution for clinical drug therapy and tissue engineering using drug microspheres with a sustained release.

## Graphical abstract

**Figure:**
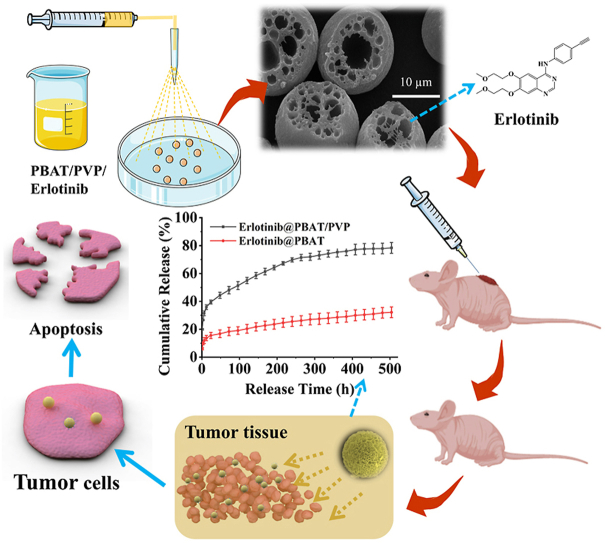

## 1 Introduction

Cancer has long been one of the major factors threatening human life. Clinical studies showed that the early diagnosis and precise treatment of cancer greatly reduce the mortality [1]. At present, the main treatments to combat cancer are surgical resection [2], chemotherapy [3], radiotherapy [4]. Also, the long-term use of chemotherapy at high doses produces strong side effects. Hence, researchers focus on improving conventional chemotherapeutic methods, such as increasing the amount of drugs in tumors despite reducing the administered drug dose, and a long-term continuous action on tumors. An effective drug delivery system may be a solution that overcomes the limitations of chemotherapy [5,6].

Sustained-release drug carriers are generally a system that enables the long-term release of drugs [7], thus reducing or even overcoming the problems of low accumulation of drugs in the tumor, poor targeting, short drug action time, and side effects caused by traditional administration methods, so that the drug can exert its effect on the lesion site for a long time [[8], [9], [10], [11], [12]]. The encapsulation of chemotherapeutic drugs in carriers made of biodegradable polymers with good biocompatibility may be a drug sustained-release system with excellent performance, with a local injection not only improving the local accumulation of drugs in the tumor, but also reducing the toxic and side effects of drugs in the body. At the same time, its sustained release allows the accumulation of the drug in the tumor, thereby improving the anti-tumor effect [13,14]. Microspheres are important sustained-release drug carriers with a large specific surface area and can efficiently load drugs. For example, Yuan [15] et al. prepared a curcumin-loaded microsphere using poly (lactic-co-glycolic acid) (PLGA) [16,17] as a shell material. The loading rate of curcumin by the microsphere is up to 48.78% ​± ​0.94, and the sustained release experiment *in vitro* showed that the microsphere is stably released within 40 days. Generally, the methods of preparing microspheres include the emulsification-evaporation, self-assembly, and electrostatic spray [[18], [19], [20]]. The electrostatic spray not only has the advantages of good encapsulation effect, narrow particle size distribution, and good dispersion, but it also easily control the particle size and surface appearance [21,22]. For example, Han [23] et al. prepared sustained-release solid lipid microspheres loaded with paclitaxel, with an average particle size of 1.76 ​μm ​± ​0.37 using the electrostatic spray method, and the solid lipid microspheres have good dispersion and spherical morphology with complete and smooth structure. This method to prepare microspheres consists of a polymer solution forming a micro-spray due to the electrostatic action in a high-voltage electrostatic field, and then, the small polymer droplets volatilize and solidify rapidly during flight to form the polymer microspheres. These microspheres prepared by this method are widely used in drug delivery, battery materials, tissue engineering, and other fields [[24], [25], [26], [27], [28], [29], [30]]. and they can load different hydrophilic and hydrophobic drugs for the clinical treatment of tumors [31,32]. For example, Ni [33] et al. prepared PLAG microspheres with an average particle size of 5.32 ​μm ​± ​0.05 coated with polydopamine and doxorubicin hydrochloride.

The method used to prepare microspheres with synthetic polymer materials is not only easier to control, but the obtained microspheres possess excellent mechanical properties and more controllable drug release compared with those prepared with natural polymer materials [[34], [35], [36]]. Microspheres prepared from PLGA, polycaprolactone [37,38] (PCL), polylactic acid [[39], [40], [41]] (PLA), and other synthetic polymers have been reported many times, while those prepared from polybutylene adipate [[42], [43], [44]] (PBAT) have not yet been reported. PBAT is a synthetic polymer with good biocompatibility and biodegradability, and the products after complete degradation are water and carbon dioxide. Its degradation rate in vivo is higher than polylactic acid, so it has attracted much attention in recent years [[45], [46], [47]]. In 2021, our research group successfully prepared hollow porous microfibers using PBAT as the skeleton material and polyethylene glycol and polyvinyl alcohol as the porogen and they were successfully loaded with an anti-cancer peptide [42], showing a good slow-release effect.

Microspheres are divided into solid microspheres, double-layer microspheres, hollow microspheres, and porous microspheres [[48], [49], [50]]. Hollow microspheres and porous microspheres have the advantages of low density, high bioavailability, and high encapsulation efficiency. Moreover, the hollows in the hollow microspheres confer them great buoyancy, good suspension, and sustained release characteristics. The bioavailability of the hollow microspheres is significantly higher than that of solid microspheres and double-layer microspheres [51]. For example, Li [52] et al. used a self-loading method to prepare hollow microspheres, which improves the encapsulation efficiency and conductivity, as well as the utilization rate of the drugs in vivo. The surface of porous microspheres has many pores, large specific surface area, low density, and strong adsorption capacity. Wang et al. [53] prepared PLGA solid microspheres, hollow microspheres, and porous microspheres, and their results showed that the encapsulation efficiency, sustained release, and final cumulative release of porous microspheres are significantly better than those of the other two microspheres. Therefore, this study combines the advantages of hollow microspheres and porous microspheres, such as low density, high bioavailability, high encapsulation efficiency, and good sustained release. The hollow degradable microspheres were prepared using PBAT and polyvinylpyrrolidone (PVP).

Lung cancer is a global health problem, and non-small cell lung cancer (NSCLC) accounts for approximately 85% of lung cancer. According to statistics, lung cancer patients increase by about 2 million every year in the world [54,55]. Erlotinib is an epidermal growth factor receptor complex kinase inhibitor (EGFR-TKI) approved by the US FDA in 2004 as a treatment to cure NSCLC. It inhibits tumor cell proliferation and promotes tumor cell apoptosis by inhibiting the transmission of the signal of epidermal growth factor receptor (EGFR) in tumor cells [56]. This work shows that PBAT/PVP hollow microspheres were successfully prepared using PBAT and PVP as raw materials by electrostatic spray technology, and then PBAT/PVP hollow microspheres were loaded with Erlotinib and used for the treatment of tumor-bearing mice, providing a theoretical basis for microsphere loaded drugs in the treatment of lung cancer.

## 2 Results and discussion

### 2.1 Preparation of PBAT microspheres

**Fig. 1:**
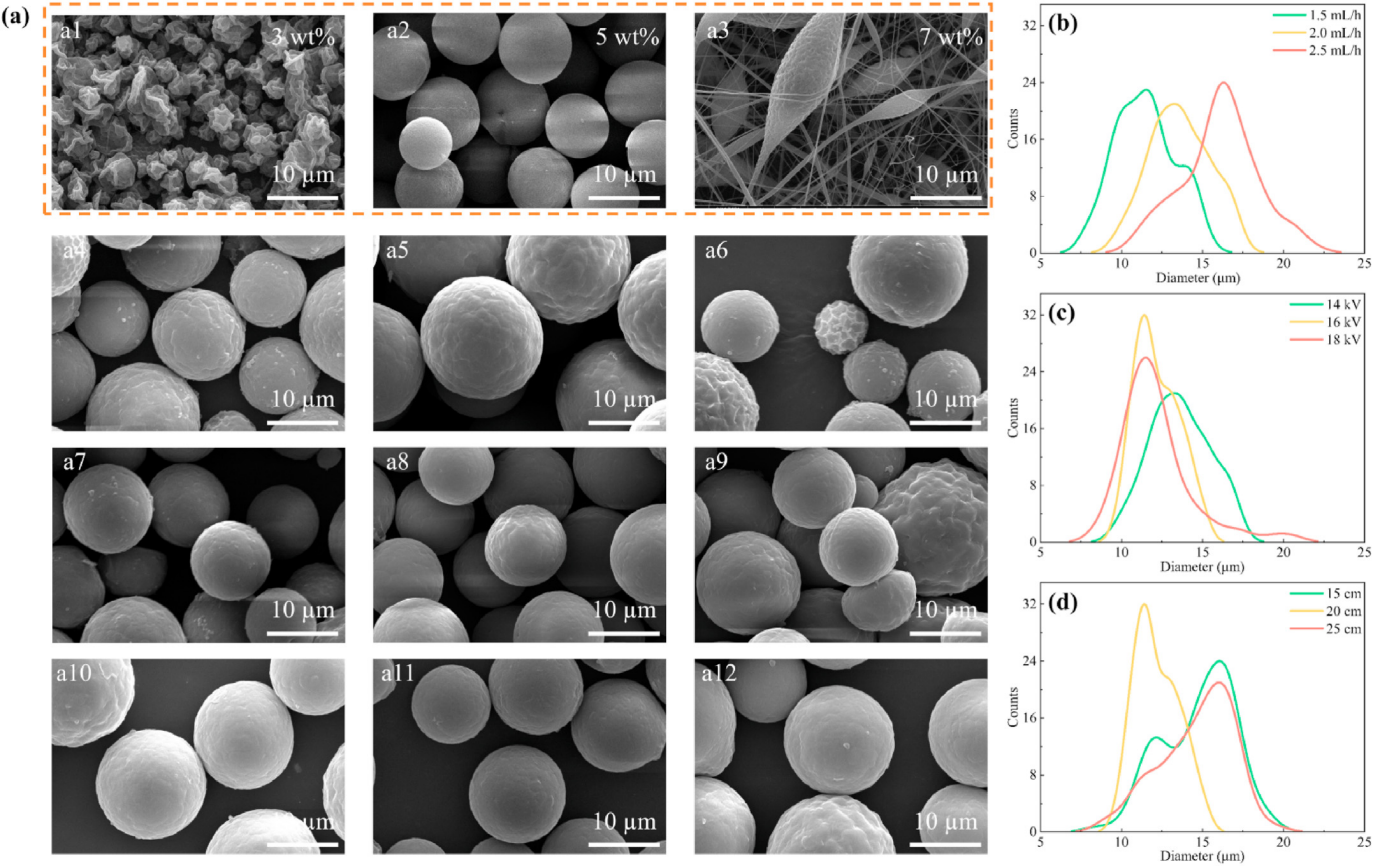
Optimization of the electrospraying process to prepare microspheres. a. a1–a3) Polymer concentrations: 3 ​wt%, 5 ​wt%, and 7 ​wt%, respectively. a4–a6) Flow rates: 1.5 ​mL/h, 2.0 ​mL/h, and 2.5 ​mL/h, respectively. a7-a9) Voltages: 14 ​kV, 16 ​kV, and 18 ​kV, respectively. a10–a12) Receiving distance: 15 ​cm, 20 ​cm, and 25 ​cm, respectively. (b), (c), and (d) show the corresponding particle size distribution.

The hollow microspheres with excellent structure were obtained by optimizing the conditions during the process, including concentration, flow rate, voltage, and receiving distance. The morphology of the microspheres was observed by scanning electron microscopy (SEM), while the particle size of the microspheres was statistically analyzed and the particles were poly dispersed using Nano Measurer 1.2 software. The results showed that the prepared samples changed from shrunk spherical to regular spherical and beaded fibers as the concentration of PBAT solution increased to 3 ​wt%, 5 ​wt%, and 7 ​wt%, respectively, as shown in Fig. 1 a1-a3.

The concentration of PBAT solution was set to 5 ​wt% to obtain microsphere samples with excellent structure in subsequent experiments. Then, the parameters of the method, such as jet flow rate, voltage, and receiving distance were optimized. The average diameters of the microspheres shown in Fig. 1 a4-a6 and b were 11.48 ​μm, 13.6 ​μm, and 16.1 ​μm ​at the flow rates of 1.5 ​mL/h, 2.0 ​mL/h, and 2.5 ​mL/h, respectively. The relative standard deviation (RSD) calculated according to Formula (1) under these conditions is shown in Table 1. RSD was 13.52% when the flow rate was 2.0 ​mL/h, indicating a more uniform particle size. Therefore, this flow rate was used to perform subsequent experiments. Then, the voltage of the prepared microspheres was optimized (Fig. 1 a7-a9 and c). The average particle size of the microspheres was 12.19 ​μm when the voltage was 16 ​kV, and the RSD was the smallest, only 11.02%. In addition, changing the receiving distance of the microspheres revealed that 20 ​cm was the optimal condition under 5 ​wt% solution concentration, a flow rate of 2.0 ​mL/h, and a voltage of 16 ​kV (Fig. 1 a10-a12 and d and Table 1). In summary, a solution concentration of 5 ​wt %, a flow rate of 2.0 ​mL/h, a voltage of 16 ​kV, and a receiving distance of 20 ​cm were chosen to obtain the most uniform particle size of PBAT microspheres. PBAT microspheres under these conditions had an average particle size of 12.19 ​μm ​± ​1.34 and an RSD of 11.02%.

**Table 1:** Diameter and relative standard deviation (RSD) of microspheres prepared under different conditions.

Variable	Parameter	Diameter (μm)	RSD (%)
External phase flow rate	1.5 ​mL/h	11.48 ​± ​1.82	15.88
	2.0 ​mL/h	13.6 ​± ​1.84	13.52
	2.5 ​mL/h	16.1 ​± ​2.33	14.47
Voltage	14 ​kV	13.6 ​± ​1.84	13.52
	16 ​kV	12.19 ​± ​1.34	11.02
	18 ​kV	12.16 ​± ​2.23	18.31
Collecting distance	15 ​cm	14.75 ​± ​2.22	15.08
	20 ​cm	12.19 ​± ​1.34	11.02
	25 ​cm	14.65 ​± ​2.25	15.38

### 2.2 Degradation of microspheres *in vitro*

**Fig. 2:**
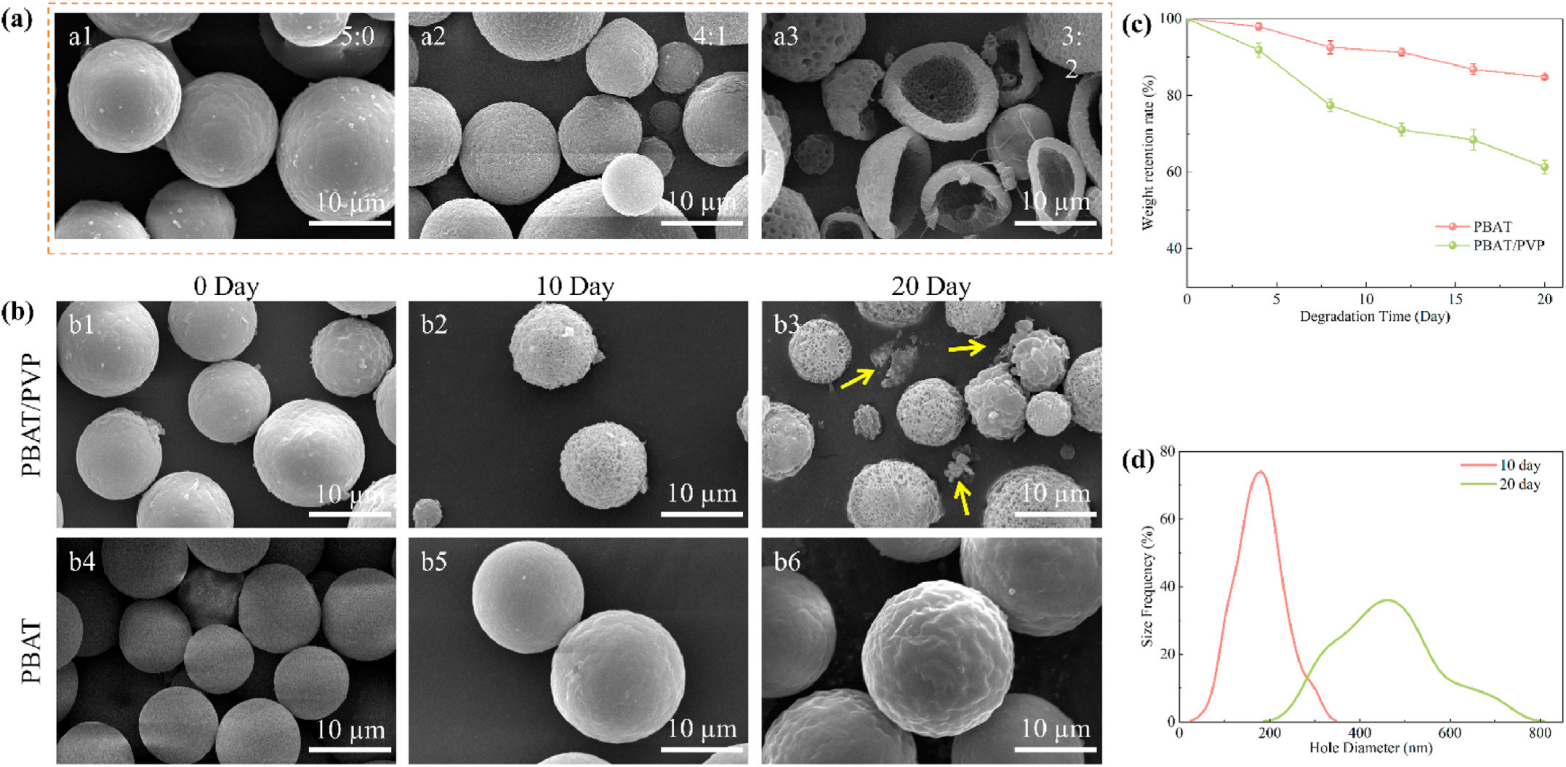
SEM images of a1) PBAT, a2) PBAT:PVP ​= ​4:1, a3) PBAT:PVP ​= ​3:2. b1-b6) SEM images of PBAT and PBAT/PVP microspheres at different degradation days. c) Short-term biodegradability of PBAT and PBAT/PVP microspheres in simulated body fluids occurring over 20 days. d) Hole size distribution of PBAT/PVP microspheres at 10 and 20 days of degradation.

Yufei Liu et al. [42] found that the degradation rate of pure PBAT fibers was 10% in 4 days, while the degradation rate of hollow porous PBAT fibers was 18% in 4 days. Inspired by this, PVP was added to PBAT to increase the degradation rate of PBAT microspheres since PVP has a faster degradation rate than PBAT and is soluble in water. According to the best preparation process to obtain PBAT microspheres described in Section 2.1, the solution concentration of 5 ​wt%, the flow rate of 2 ​mL/h, the voltage of 16 ​kV, and the receiving distance of 20 ​cm were selected as the conditions for preparing PBAT/PVP microspheres with uniform particle size, and the differences in the characteristics of microspheres prepared by different ratios of PBAT and PVP were evaluated. Fig. 2, a1-a3 show SEM images of PBAT and PVP with different mass ratios. When PBAT:PVP ​= ​5:0, 4:1, or 3:2, the microspheres did not have any hole on the surface, a small hole on the surface, or a bowl-like structure with holes on the surface, respectively. Therefore, the ratio PBAT:PVP ​= ​4:1 with better balling and uniform pore distribution was chosen to increase the degradation performance of the microspheres. PBAT/PVP microspheres with an average particle size of 11.14 ​μm ​± ​1.74 and RSD of 15.65% (Fig. 2 a2) were prepared under the above protocol and PVP addition. The PBAT/PVP microspheres and PBAT microspheres with different degradation days were removed from the PBS to further confirm that the addition of PVP increased the degradation rate of the microspheres, and the morphology was observed by SEM after drying (Fig. 2 b1-b6). The results showed that the holes on the surface of PBAT/PVP microspheres became larger on the 10th day of degradation, and they became even larger on the 20th day, and the debris appeared (Fig. 2 b3 yellow arrow). No evident change was observed on the surface of pure PBAT microspheres. Moreover, the weight retention curve (Fig. 2c) showed that the degradation rate of PBAT microspheres in PBS for 20 days was only 15.18% ​± ​0.52, while the degradation rate of PBAT/PVP microspheres was 38.68% ​± ​1.71. The distribution of the surface pore size of PBAT/PVP microspheres degraded for 10 days and 20 days was further counted, as shown in Fig. 2d used to Nano Measurer 1.2 software and they were 0.18 ​μm ​± ​0.05 and 0.46 ​μm ​± ​0.11, respectively, indicating that the surface pore size of PBAT/PVP microspheres increased with the increase of the degradation time.

### 2.3 Drug loading and *in vitro* release

**Fig. 3:**
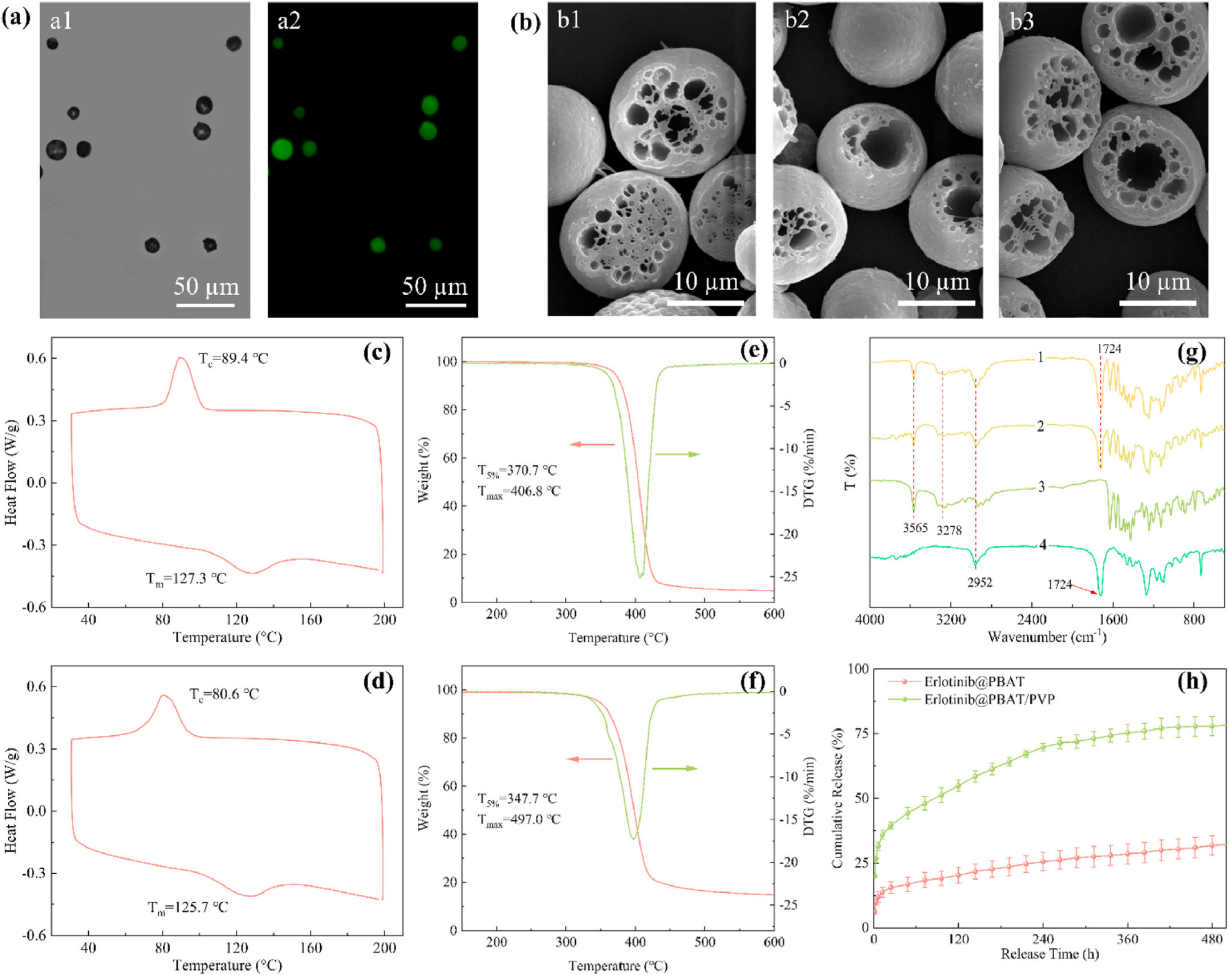
a1 and a2) Inverted fluorescence microscope image of Erlotinib@PBAT/PVP microsphere. b1-b3) Cross-sectional SEM images of PBAT microspheres, Erlotinib@PBAT microspheres and Erlotinib@PBAT/PVP microspheres, respectively. c) and d) DSC curves of PBAT microspheres and Erlotinib@PBAT/PVP microspheres, respectively. e and f) TGA curves of PBAT microspheres and Erlotinib@PBAT/PVP microspheres, respectively. g) FTIR spectra of PBAT, erlotinib, Erlotinib@PBAT, and Erlotinib@PBAT/PVP (1 ​= ​Erlotinib@PBAT/PVP, 2 ​= ​Erlotinib@PBAT, 3 ​= ​erlotinib, 4 ​= ​PBAT). h) Drug release curve of Erlotinib@PBAT and Erlotinib@PBAT/PVP.

The drug-loaded microspheres were prepared by adding erlotinib to the solution of 8.5% PBAT and PVP mass. Then, the Erlotinib@PBAT microspheres and Erlotinib@PBAT/PVP microspheres were dissolved, and the UV absorbance of the solution was measured. The drug loading rate and encapsulation efficiency were calculated by (2), (3). The drug loading rates of Erlotinib@PBAT microspheres and Erlotinib@PBAT/PVP microspheres were 5.38% ​± ​1.53 and 5.21% ​± ​1.05, respectively, and the encapsulation efficiency was 92.13% ​± ​2.76 and 91.8% ​± ​5.16, respectively. Since the erlotinib molecule has green fluorescence, the Erlotinib@PBAT/PVP microspheres in bright and green fluorescence fields shown in Fig. 3 a1 and **a2** respectively, were observed under an inverted fluorescence microscope, and indicated that erlotinib was loaded onto the microspheres. The cross-section sample of the Erlotinib@PBAT/PVP microspheres was prepared by the method described in the paragraph 4.5 and was observed under SEM. The cross sections of PBAT microspheres and Erlotinib@PBAT/PVP microspheres indicated that both had a hollow structure with multiple pores (Fig. 3 b1-b3). This indicated that the addition of PVP and drug loading did not change the internal structure of the microspheres. Fig. 3c and d shows the differential scanning calorimetry curves of PBAT microspheres and Erlotinib@PBAT/PVP microspheres, respectively. The diagram revealed that the melting temperature (T_m_) and crystallization temperature (T_c_) of the drug-loaded microspheres with PVP were decreased, which was due to the incorporation of PVP and erlotinib. Fig. 3e and f shows the TGA curves of PBAT microspheres and Erlotinib@PBAT/PVP microspheres, respectively. The maximum degradation temperature of the drug-loaded microspheres with PVP also decreased, which was the same as the conclusion obtained using the differential scanning calorimeter (DSC). Fig. 3g is the infrared spectra of erlotinib, PBAT, Erlotinib@PBAT microspheres, and Erlotinib@PBAT/PVP microspheres; 3565 ​cm^−1^ was the stretching vibration of N–H in erlotinib; 3278 ​cm^−1^ was the stretching vibration of alkynyl hydrogen [57], 2952 ​cm^−1^ was the C–H stretching vibration absorption peak; and 1724 ​cm^−1^ was the C = O stretching vibration absorption peak in PBAT and PVP [58]. The figure showed that erlotinib was successfully loaded onto the microspheres. The release of the drug by Erlotinib@PBAT and Erlotinib@PBAT/PVP microspheres in PBS lasted for 21 days, as shown in the curves of Fig. 3h. The cumulative release amount of the two microspheres at 21 days was 78.55% ​± ​3.9 and 31.81% ​± ​3.85, respectively, revealing that the release rate of Erlotinib@PBAT/PVP microspheres was significantly higher than that of Erlotinib@PBAT microspheres. This was due to the fact that the addition of PVP made the microspheres easier to degrade, thus accelerating the release of drugs, which is consistent with the degradation effect of Fig. 2b.

### 2.4 Cytocompatibility and *in vitro* anti-tumor test

**Fig. 4:**
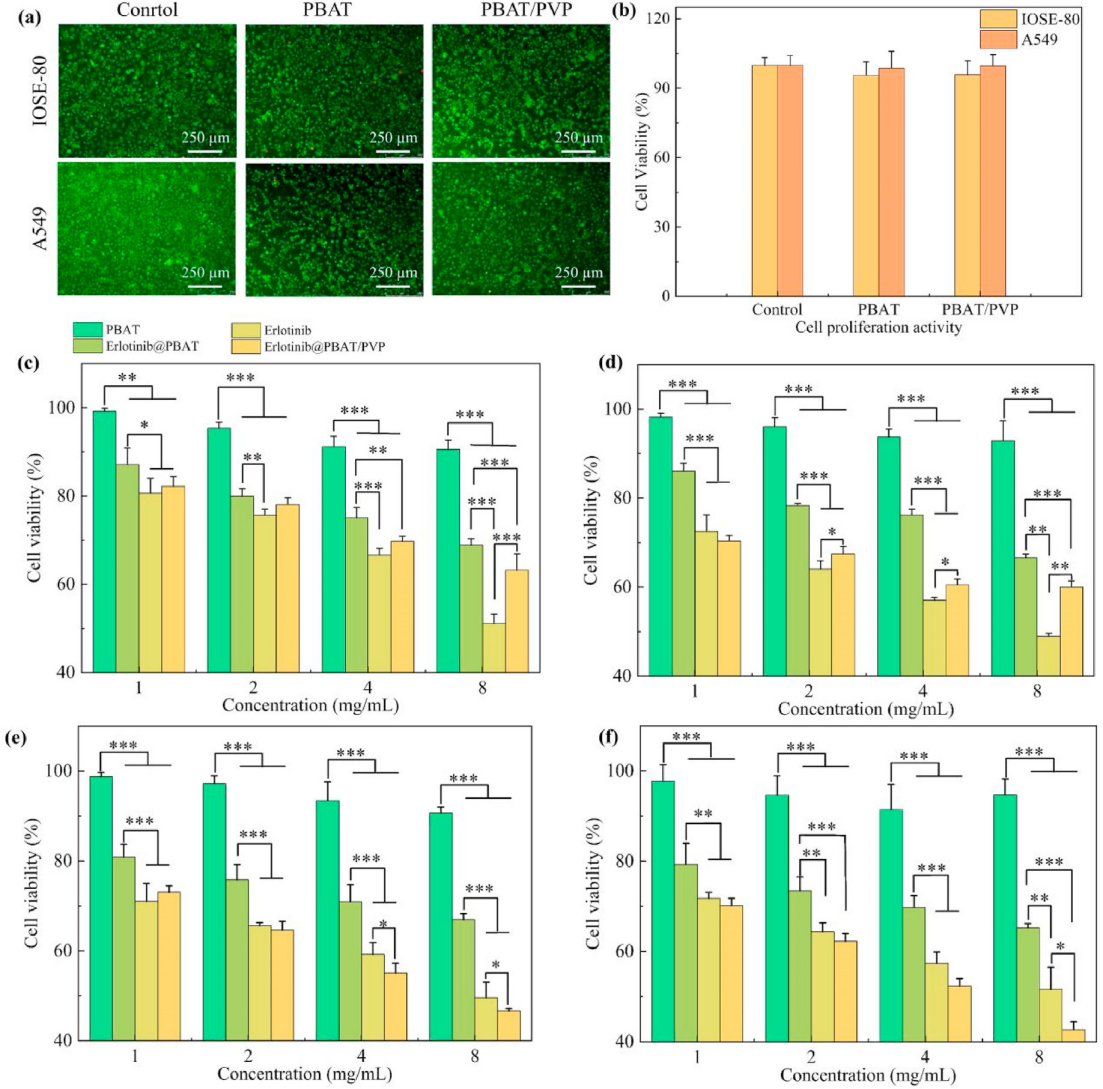
*In vitro* cell viability of IOSE-80 and A549 ​cells. (a) Fluorescence images showing the viability of IOSE-80 and A549 ​cells following the treatment with 4 ​mg/mL Erlotinib@PBAT and Erlotinib@PBAT/PVP microspheres for 24 ​h. Green calcein AM fluorescence and red PI fluorescence indicate live and dead cells, respectively. (b) The coculture of IOSE-80 and A549 ​cells on 24 ​h after treatment with the Erlotinib@PBAT and Erlotinib@PBAT/PVP microspheres. (c–f) A549 ​cells incubated with different concentrations of Erlotinib@PBAT and Erlotinib@PBAT/PVP microspheres for 24 ​h, 48 ​h, 72 ​h, and 96 ​h (∗*p* ​< ​0.05, ∗∗*p* ​< ​0.01, and ∗∗∗*p* ​< ​0.001).

The blank PBAT and PBAT/PVP microspheres were co-incubated with ovarian surface epithelial cells IOSE-80 and lung cancer A549 at a concentration of 4 ​mg/mL to assess the compatibility of the two microspheres with the cells. Green calcein AM fluorescence and red PI fluorescence represent living cells and dead cells, respectively (Fig. 4a). These two microspheres did not have any effect on cell growth (Fig. 4b); thus, they were used for subsequent experiments. Fig. 4c–f shows the viability of A549 ​cells incubated with different concentrations of erlotinib, PBAT, Erlotinib@PBAT, and Erlotinib@PBAT/PVP microspheres at 24 ​h, 48 ​h, 72 ​h, and 96 ​h. When A549 ​cells were treated with erlotinib for 24 ​h and 48 ​h, the inhibitory effect of erlotinib on cell viability was stronger than that of drug-loaded microspheres. This may be due to the fact that A549 ​cells were exposed to high concentrations of erlotinib over a period of time, resulting in more apoptosis of A549 ​cells. However, Erlotinib@PBAT/PVP microspheres had a stronger inhibitory effect on A549 ​cell viability than erlotinib at the same concentration at 72 ​h and 96 ​h (*p* ​< ​0.05). This may be due to the sustained release effect of Erlotinib@PBAT/PVP microspheres, which maintained a high drug concentration for a long time that inhibit the viability of A549 ​cells. In addition, the uptake of Erlotinib@PBAT/PVP microspheres by cells might be increased, thereby promoting the apoptosis of A549 ​cells.

### 2.5 *In vivo* tumor xenograft experiment

**Fig. 5:**
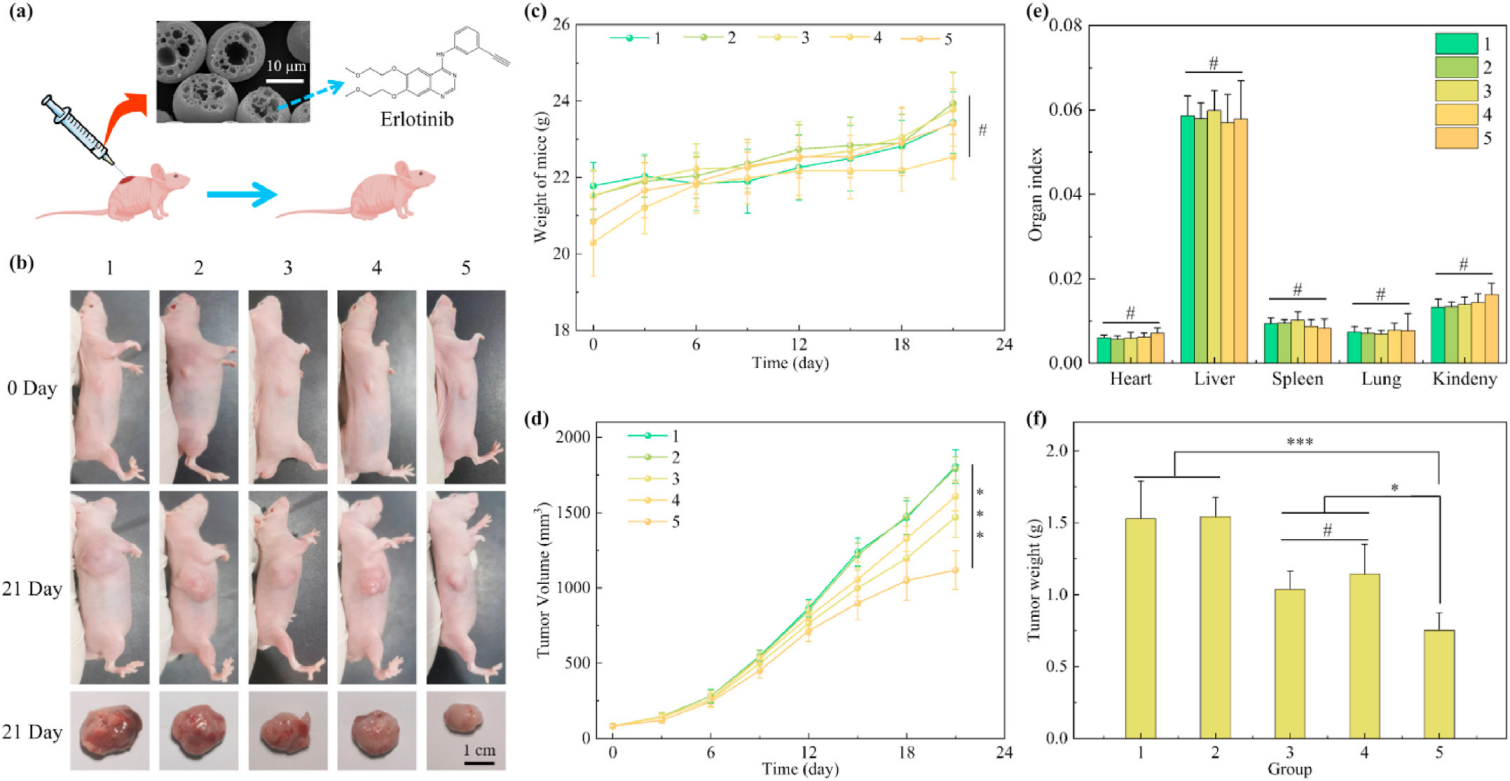
PBAT, Erlotinib, Erlotinib@PBAT and Erlotinib@PBAT/PVP were used to treat tumors in mice. a) Schematic diagram of tumor xenograft experiment in vivo. b) Photos of A549 tumor-bearing mice and excised A549 solid tumors from different treatment groups at day 21. c) Body weight changes. d) Organ index. e) Tumor volume. f) The weight of tumor tissues. (1 ​= ​PBS; 2 ​= ​PBAT; 3 ​= ​Erlotinib; 4 ​= ​Erlotinib@PBAT; 5 ​= ​Erlotinib@PBAT/PVP) (∗*p* ​< ​0.05, ∗∗*p* ​< ​0.01, ∗∗∗*p* ​< ​0.001 and #*p* ​> ​0.05 compared with the Erlotinib@PBAT/PVP group at the corresponding time point).

The successful tumor-bearing nude mouse model was treated once every 3 days according to the grouping described in the paragraph 4.10 of the experimental section, and the body weight and tumor volume of the mice were recorded every two days until the end of the treatment. Fig. 5a is a diagram of the animal experiment protocol. Fig. 5b shows a picture of the beginning and end of the treatment on mice with successful modeling, as well as a picture of the tumor at the time of dissection. No significant difference in tumor size was found between the PBS group and PBAT group, indicating that the injection of PBAT microspheres into the mouse tumor did not affect tumor growth, which is consistent with the results of cell compatibility experiments. Erlotinib, Erlotinib@PBAT, and Erlotinib@PBAT/PVP exerted different therapeutic effects on mouse tumors. Indeed, the photos of mice after dissection of Fig. 4b showed that the tumors of mice treated with Erlotinib@PBAT/PVP was the smallest and significantly smaller than that of the mice treated with erlotinib, indicating that Erlotinib@PBAT/PVP exerted a better anti-tumor effect than erlotinib, which was consistent with the *in vitro* anti-tumor results shown in Fig. 4c–f. The tumor size of the mice treated with Erlotinib@PBAT was between that of the erlotinib mice and PBAT mice, indicating that the intratumoral injection of Erlotinib@PBAT/PVP microspheres resulted in a certain therapeutic effect, although the therapeutic effect was weaker than that of a direct injection of erlotinib. Fig. 5c shows the curve of the weight change in each group of mice from the beginning of the treatment to the end. The body weight in each group increased to varying degrees, although with no significant difference among the groups. The organ index in each group of mice was calculated according to the body weight and the weight of the main organs (heart, liver, spleen, lung, and kidney) after dissection. The results in Fig. 5d showed no significant difference in the organ index of mice in each group. The changes in tumor volume from the beginning of the treatment to the day of dissection in each group of mice shown in Fig. 5e revealed that the tumor volume of the Erlotinib@PBAT/PVP mice after 21 days of treatment was significantly different from that of other groups (*p* ​< ​0.001), which was consistent with the results of tumor dissection shown in Fig. 5b. The tumor weight of the mice in each group after dissection also showed that it was less in the Erlotinib@PBAT/PVP group than in the other groups (Fig. 5f). Fig. 5f also showed that the tumor mass of mice in the PBS group and PBAT group was comparable, indicating that intratumoral injection of PBAT microspheres did not affect the tumor growth of mice. Erlotinib, Erlotinib@PBAT, and Erlotinib@PBAT/PVP exerted certain therapeutic effects. Among them, the tumor growth of mice in the Erlotinib@PBAT/PVP group was the smallest. The average tumor weight of the mice in this group was only 0.75 ​g ​± ​0.12 after 21 days of treatment. The tumor weight of Erlotinib@PBAT/PVP group was smaller than that of PBS group and PBAT group (*p* ​< ​0.001). The tumor weight of the Erlotinib@PBAT/PVP group was significantly lower compared to that of the other two groups (*p* ​< ​0.05), indicating that the injection of Erlotinib@PBAT/PVP microspheres with the sustained release effect had the best therapeutic effect. The tumor weight of the mice in the Erlotinib@PBAT group was lower than that in the PBS group and PBAT group, but slightly higher than that in the erlotinib group, with no significant difference between the tumor weight of the mice in the Erlotinib@PBAT group and that in the erlotinib group. These results might be due to the fact that pure PBAT had too strong coating effect on erlotinib, so that the release of Erlotinib was too slow, resulting in a slightly worse therapeutic effect of Erlotinib@PBAT than that of the direct intratumoral injection of erlotinib but better than that of PBS and PBAT. This was also consistent with the *in vitro* anti-tumor effect and the results shown in the tumor photos of each group of mice in Fig. 5b.

**Fig. 6:**
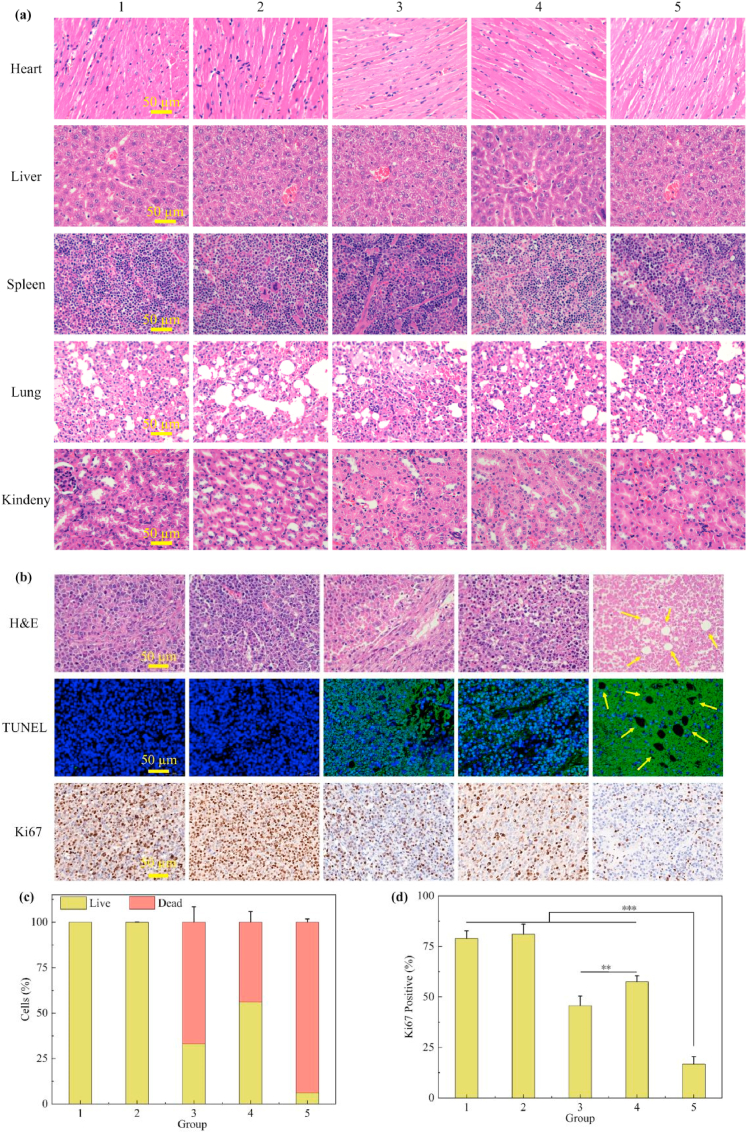
a) H&E staining images of the main mouse organs. b) H&E, TUNEL, and Ki67 staining images of tumor tissues in mice of each group. c) Statistical results of TUNEL staining. d) Ki67 protein expression in the tumors of mice in each group detected by immunohistochemistry. (1 ​= ​PBS; 2 ​= ​PBAT; 3 ​= ​erlotinib; 4 ​= ​Erlotinib@PBAT; 5 ​= ​Erlotinib@PBAT/PVP) (∗*p* ​< ​0.05, ∗∗*p* ​< ​0.01, and ∗∗∗*p* ​< ​0.001).

The mice in each group were euthanized and dissected at the end of the treatments. The staining of main organs (heart, liver, spleen, lung and kidney) shown in Fig. 6a showed that the organs of mice in each group were intact, the cells were closely arranged, and no obvious lesions were observed, indicating that the microspheres had no significant effect on the organs of tumor-bearing mice, which was consistent with the results of organ index.

The results of H&E, *in situ* terminal deoxynucleotidyl transferase-mediated UTP end labeling (TUNEL), and immunohistochemical analysis on the tumors of each group shown in Fig. 6b revealed almost no apoptosis of tumor cells in the two groups treated with PBS and PBAT microspheres. The tumor cells in the erlotinib group and the Erlotinib@PBAT group showed a certain degree of apoptosis and some gaps, while the proportion of tumor cell apoptosis in the erlotinib group was more evident, and the gaps among tumor cells were larger. The apoptosis rate of tumor cells in mice treated with Erlotinib@PBAT/PVP microspheres was the highest, accompanied by incomplete cell morphology, nuclear fragmentation, sparse cell arrangement, and evident voids (Fig. 6b yellow arrow). Similarly, the TUNEL staining results (Fig. 6b and c) also showed the highest number of apoptotic cells and the lowest number of tumor cells in the Erlotinib@PBAT/PVP group cells and voids were also present (Fig. 6b yellow arrow) compared with the other four groups. The tumor cells in the PBS group and the PBAT group were not affected and were in a normal growth state. The growth of tumor cells in the erlotinib group and Erlotinib@PBAT group was affected to a certain extent, and the effect on the Erlotinib@PBAT group was between the untreated group and the erlotinib group, which was the same as the H&E staining results. The abnormal growth of tumor cells in the Erlotinib group was less than that in the Erlotinib@PBAT group, probably because the pure PBAT encapsulated Erlotinib in a stronger manner, resulting in a lower antitumor activity of Erlotinib@PBAT than Erlotinib, which is described above in the *in vitro* degradation, in the release experiments, *in vitro* antitumor activity experiments and tumor growth in Fig. 5. In addition, the expression of Ki67 protein in tumor tissue sections of mice in each group was counted by Image J software. The expression of Ki67 protein shown in Fig. 6d revealed that it was the smallest in the Erlotinib@PBAT/PVP group and it was extremely significant compared with its expression in the other groups (*p* ​< ​0.001), while it was highly expressed in the PBS group and the PBAT group. The erlotinib group and Erlotinib@PBAT/PVP group had a certain degree of expression of the Ki67 protein, and its expression in the Erlotinib group was significantly higher than that in the Erlotinib@PBAT group (*p* ​< ​0.01), indicating that the sustained release effect of Erlotinib@PBAT/PVP microspheres strengthened the inhibition of tumor cell proliferation and improved the anti-tumor activity.

## 3 Conclusion

In conclusion, biodegradable PBAT/PVP microspheres with an average particle size of 11.14 ​μm ​± ​1.74 and an RSD of 15.65% were prepared by electrostatic spray technology. The hollow structure of the PBAT/PVP microspheres with drug release function conferred them a biodegradable property and microspheres degradation was 38.68% ​± ​1.71 in 21 days, while the degradation of the pure PBAT microspheres was only 15.18% ​± ​0.52. Then, erlotinib was successfully loaded on the two microspheres, and the loading rate of the two microspheres was comparable. The *in vitro* release of the drug-loaded microspheres showed that the drug release efficiency of Erlotinib@PBAT/PVP was much higher than that of Erlotinib@PBAT. The cumulative release rates of Erlotinib@PBAT/PVP and Erlotinib@PBAT microspheres after three weeks were 78.55% ​± ​3.9 and 31.81% ​± ​3.85, respectively. The treatment of A549 ​cells with blank microspheres showed that the prepared PBAT microspheres and PBAT/PVP microspheres had good biocompatibility. The *in vitro* anti-tumor studies showed that Erlotinib@PBAT/PVP had a stronger inhibitory effect on A549 ​cells after 72 ​h of co-incubation than the direct addition of erlotinib at a comparable dose. Animal experiments further demonstrated that the tumors in mice treated with Erlotinib@PBAT/PVP microspheres in the same treatment cycle were smaller than those in mice treated with erlotinib at the same dose. This study demonstrated that the drug delivery system continuously and stably released the drug, maintaining a high drug concentration in the tumor for a long time, achieving better therapeutic effects. Thus, this approach might have great development potential and might represent a new strategy in clinical cancer treatment.

## 4 Experimental section

### 4.1 Materials

PBAT (*M*_n_ ​= ​20 ​k) was purchased from Jinhui Zhaolong High Technology Co., Ltd. (Xiaoyi City, China). PVP (*M*_n_ ​= ​8 ​k) was purchased from Shanghai Aladdin Biochemical Technology Co., Ltd. (Shanghai, China) and directly used without treatment. Dichloromethane (CH_2_Cl_2_) was purchased from Chengdu Kelong Chemical Reagent Factory (Chengdu, China). Erlotinib was purchased from Shanghai Bide Pharmaceutical Technology Co., Ltd. (Shanghai, China).

### 4.2 Cell lines and culture medium

Human ovarian epithelial cells (IOSE-80) and human non-small cell lung cancer cell line (A549) were obtained from the Key Laboratory of Natural Product Chemistry, Chinese Academy of Sciences, Guizhou Province (Guiyang, China). Both cells were cultured in Dulbecco's Modified Eagle Medium (DMEM) containing 10% fetal bovine serum (FBS) and 100 U/mL penicillin and streptomycin (Gibco, USA). All cells were incubated at 37 ​°C, 5% CO_2,_ under 100% humidity.

### 4.3 Animals

Thirty BALB/c-nu male nude mice (20 ​± ​2 ​g), 5 week old were randomly divided into five groups of six mice in each group, to ensure that no less than three mice in each group were used for a successful modeling and subsequent experiments, and they were kept at 37 ​°C and 55% humidity in the animal center. All animal experiments were approved by the Protection and Use Committee of Guizhou Medical University and conducted according to the Guidelines for the Protection and Use of Experimental Animals.

### 4.4 Preparation of microspheres

The influence of PBAT dichloromethane solution at different concentrations (3 ​wt%, 5 ​wt%, and 7 ​wt%) on the balling effect was investigated. Then, the effects of different flow rates, voltage, and receiving distance on the morphology and particle size of microspheres were assessed. In addition, a certain proportion of PVP was added to the solution to increase the degradation rate of microspheres, and the degradation effect of microspheres prepared using PBAT and PVP with different mass ratios (5:0, 4:1, and 3:2) was investigated.

PBAT and PVP solid particles were added to dichloromethane, and place under a magnetic stirrer at room temperature for 4 ​h. The stirring was used to prevent the volatilization of dichloromethane as much as possible, to obtain different mass concentrations of the solution. A 10 ​mL syringe (KDS100, NanoNC, Korea) connected to a 20 ​G needle was then filled with the solution, and the solution was pushed out from the syringe using a syringe pump (Longer pump LSP01-1 ​A) at a certain speed. Receive the product under the needle in a petri dish containing ethanol. In this process, the needle and the high voltage device (Dongwen High Voltage, Tianjing, China) were connected by wires. Erlotinib was added to the above solution to prepare drug-loaded microspheres.

### 4.5 Characterization of microsphere morphology

The morphology of the microspheres was photographed using SEM (QUANTA 250FEG, FEI, America) under the working voltage of 20.00 ​kV, and several different areas were in each sample were randomly selected and observed at specific magnification. Then, Nano Measurer 1.2 software was used to analyze SEM images, measure the particle size of at least 100 microspheres for each sample, and a particle size distribution map was plotted. The polydispersity of microspheres was measured using formula (1) according to previous reports [59,60].

(1) $$RSD=\frac{1}{\bar{d}}{\left[\sum_{i=1}^{n}\frac{{\left(d-\bar{d}\right)}^{2}}{n-1}\right]}^{\frac{1}{2}}\times 100\%$$

where $d_{i}$ is the diameter of the microspheres, $\bar{d}$ is the arithmetic mean of the microspheres, and n is the total number of microspheres (n ​≥ ​100). The polydispersity index of the microspheres is defined as the RSD, that is, the ratio of the standard deviation to the average particle size, which is used to evaluate the polydispersity of microspheres. The smaller the RSD, the more uniform the particle size.

The prepared microspheres were dispersed in deionized water, then subjected to ultrasonic dispersion, rapid freezing with liquid nitrogen, sectioning, sublimation for 5–10 ​min to expose the cross-section, gold spraying, and finally SEM images were observed.

### 4.6 Characterization of erlotinib-loaded microspheres

The standard curve of the UV absorption of Erlotinib was plotted using a UV visible spectrophotometer (Cary 60, Agilent, USA). Erlotinib@PBAT and Erlotinib@PBAT/PVP microspheres, 10 ​mg each, were fully dissolved in 5 ​mL dichloromethane. One mL of this solution and 10 ​mL acetonitrile-PBS solution (1:1, V/V) were added in a 15 ​mL centrifuge tube and centrifuged at 2500 ​rpm for 3 ​min, then the supernatant was collected to measure the absorbance at 246 ​nm, and the drug loading amount and drug loading efficiency was calculated. The standard curve of erlotinib was used to determine the concentration of erlotinib in the supernatant. Then, the loading capacity (LC) and encapsulation efficiency (EE) of erlotinib were calculated according to the following formulas:

(2) $$\text{LC}\left(\text{wt}\%\right)=\frac{\text{mass}\;\text{of}\;\text{loaded}\;\text{drug}}{\text{mass}\;\text{of}\;\text{drug}\;\text{loadedmicorpheres}}\times 100\%$$

(3) $$\text{EE}\left(\text{wt}\%\right)=\frac{\text{mass}\;\text{of}\;\text{loaded}\;\text{drug}}{\text{mass}\;\text{of}\;\text{feeding}\;\text{drug}}\times 100\%$$

In addition, the prepared microspheres were scanned 32 times by Fourier transform infrared spectroscopy (FTIR, Nicolet 6700, Nicolet, USA) with a resolution of 4 ​cm^−1^ in the wave number range of 400–4000 ​cm^−1^. The thermal degradation of PBAT microspheres and Erlotinib@PBAT/PVP microspheres was obtained under the protection of nitrogen (the flow rate of the protective gas was 20 ​mL/min, and the flow rate of the purging gas was 50 ​mL/min) using a NETZSCH instrument TG 209 F1 (NETZSCH, Cerb, Germany) from 30 ​°C to 600 ​°C at a heating rate of 10 ​°C/min. An appropriate amount of PBAT microspheres and Erlotinib@PBAT/PVP microspheres was heated from 35 ​°C to 200 ​°C at the rate of 10 ​°C/min with DSC (Q10, TA, USA) in a nitrogen atmosphere with a flow rate of 50 ​mL/min for 5 ​min, and then cooled to 35 ​°C at the rate of 10 ​°C/min for characterization and analysis.

Since erlotinib has green fluorescence [61], the erlotinib-loaded microspheres were observed under an inverted fluorescence microscope (DMi8, Leica, Germany) to determine the intuitive loading of erlotinib.

### 4.7 *In vitro* degradation of microspheres

An appropriate amount of PBAT microspheres and PBAT/PVP microspheres were placed into a centrifuge tube containing 10 ​mL phosphate buffer solution (PBS) that was shaken at 37 ​°C at the speed of 100 ​rpm. An appropriate number of solid particles was dried on the 10th and 20th day, the dried samples were attached to a metal disk with conductive adhesive, sprayed with gold, and the morphology of the microspheres was observed using SEM. In addition, the short-term biodegradability of microspheres was characterized by placing PBAT and PBAT/PVP microspheres in PBS; one group was taken out every 4 days, washed, dried, and weighed; the mass retention was calculated; and the degradation rate curve was plotted.

### 4.8 Drug release *in vitro*

Erlotinib@PBAT and Erlotinib@PBAT/PVP microspheres, 100 ​mg each, were dispersed and immersed in a centrifuge tube containing 5 ​mL PBS. The centrifuge tube was shaken at 37 ​°C and a speed of 100 ​rpm. The centrifuge tube was taken out at a predetermined time interval and 1 ​mL supernatant was mixed with 1 ​mL PBS and 2 ​mL acetonitrile. The absorbance was detected at 246 ​nm by an ultraviolet–visible spectrophotometer to determine the amount of erlotinib released. After sampling, 1 ​mL fresh PBS was added to the centrifuge tube.

### 4.9 Cytocompatibility assay

IOSE-80 and A549 ​cells were seeded into a 24-well plate at a density of $2\times 10^{4}$ cells per well. After cell adhesion, 4 ​mg/mL PBAT/PVP and PBAT/PVP microspheres were added, incubated for 24 ​h, and the cells were washed with PBS three times. Then, calcein AM and propidium iodide (PI) solution were added and incubated for 15 ​min, and then the staining was observed under the inverted fluorescence microscope.

A549 ​cells were cultured in 96 well plates at the seeding density of 5 ​× ​10^3^ ​cells per well. After cell adhesion, Erlotinib@PBAT and Erlotinib@PBAT/PVP microspheres at different concentrations (1 ​mg/mL, 2 ​mg/mL, 4 ​mg/mL, and 8 ​mg/mL) were added to the cells and incubated at 37 ​°C. Cell viability was measured at 24 ​h, 48 ​h, 72 ​h, and 96 ​h.

### 4.10 *In vivo* tumor xenograft experiment

Under aseptic conditions, 0.1 ​mL cell suspension containing approximately 5 ​× ​10^6^ A549 ​cells was subcutaneously injected into the right flanks of BABL/c-nu mice to establish a nude mouse model. After the tumor volume reached 50 ​mm^3^, the tumor-bearing mice with a successful model were randomly divided into five groups: PBS, PBAT, erlotinib, Erlotinib@PBAT, and Erlotinib@PBAT/PVP, which were injected inside the tumor every two days at the dose of 5 ​mg/kg erlotinib for each mouse. The mice were treated for three consecutive weeks, and the body weight and tumor volume of the mice were recorded every 3 days. The tumor volume was measured using a vernier caliper, and calculated as V = (a ​× ​b^2^)/2, where a is the length of the tumor, and b is the width perpendicular to a. One mouse in each group was randomly selected and photographed after 21 days of treatment, and then, the mice were euthanized, dissected and the following organs (heart, liver, spleen, lung, kidney, and tumor) were collected and preserved in formalin solution. Sections of each organ and tumor were cut and stained with hematoxylin/eosin (H&E). dUTP nick-end labeling (TUNEL) was performed on the tumor using an *in situ* cell death detection kit, and Ki67 polyclonal antibody immunostaining (Abcam, Cambridge) was used to further study the anti-tumor effect of microspheres. Stained tissues were observed and imaged using a light microscope (BA210, Motic, China).

### 4.11 Statistical analysis

Statistical analysis was performed using SPSS. Unless otherwise specified in this article, all experiments were performed in at least 3 parallel groups, and the results were expressed as mean ​± ​standard deviation (mean ​± ​SD). One-way analysis of variance was used to determine whether the results were statistically significant. When p ​> ​0.05, there was no significant difference, indicated by #. When 0.05 < *p* ​< ​0.01, there was a significant difference, expressed as ∗, when 0.01 < *p* ​< ​0.001 and 0.001 < *p*, the difference was extremely significant, expressed as ∗ ∗ and ∗ ∗ ∗ respectively.

## Author statement

Chuan Xie**,**Yufei Liu**,**Xi Zeng**,**Heng Luo: Conceptualization, Methodology, Software Chuan Xie**,**Qinqin Xiong**,**Yuanzhi Wei**,**Xin Li**,**Jiajun Hu**,**Min He**,**Shinan Wei**,**Jia Yu**,**Sha Cheng**,**Mashaal Ahmad: Data curation, Writing – original draft. Chuan Xie**,** Sihai Luo**:** Visualization, Investigation. Yufei Liu**,** Xi Zeng**,** Jie Yu**,** Heng Luo**,** Sihai Luo: Supervision and Funding: All athours contrituted to Writing- Reviewing and Editing

## Declaration of competing interest

The authors declare that they have no known competing financial interests or personal relationships that could have appeared to influence the work reported in this paper.

## Data Availability

Data will be made available on request.

## References

[1] Siegel R.L., Miller K.D., Wagle N.S. (2023). Cancer statistics, 2023. CA: CA Cancer J. Clin..

[2] Chiang A., Yu K., Chao K. (2011). The incidence of isolated para-aortic nodal metastasis in completely staged endometrial cancer patients. Gynecol. Oncol..

[3] Jing Y., Xiong X., Ming Y. (2018). A multifunctional micellar nanoplatform with pH-triggered cell penetration and nuclear targeting for effective cancer therapy and inhibition to lung metastasis. Adv. Healthc. Mater..

[4] Her S., Jaffray D.A., Allen C. (2017). Gold nanoparticles for applications in cancer radiotherapy: mechanisms and recent advancements. Adv. Drug Deliv. Rev..

[5] Chew S.A., Danti S. (2017). Biomaterial-Based implantable devices for cancer therapy. Adv. Healthc. Mater..

[6] Lan X., She J., Lin D. (2018). Microneedle-mediated delivery of lipid-coated cisplatin nanoparticles for efficient and safe cancer therapy. ACS Appl. Mater. Interfaces.

[7] Ruan L., Su M., Qin X. (2022). Progress in the application of sustained-release drug microspheres in tissue engineering. Mater.Today.Bio..

[8] Bhise N.S., Ribas J., Manoharan V. (2014). Organ-on-a-chip platforms for studying drug delivery systems. J. Contr. Release.

[9] Fonte P., Reis S., Sarmento B. (2016). Facts and evidences on the lyophilization of polymeric nanoparticles for drug delivery. J. Contr. Release.

[10] Chen C., Liu Y., Sun L. (2019). Antibacterial porous microcarriers with a pathological state responsive switch for wound healing. ACS Appl. Bio Mater..

[11] Liu Y., Li M., Yang F. (2017). Magnetic drug delivery systems. Sci.China Mater.

[12] Liang J., Liu B. (2016). ROS-responsive drug delivery systems. Bioeng. Transl. Med..

[13] Yuan S., Lei F., Liu Z. (2015). Coaxial electrospray of curcumin-loaded microparticles for sustained drug release. PLoS One.

[14] Dwivedi P., Yuan S., Han S. (2018). Core-shell microencapsulation of curcumin in PLGA microparticles: programmed for application in ovarian cancer therapy. Artif. Cells, Nanomed. Biotechnol..

[15] Yuan S., Lei F., Liu Z. (2015). Coaxial electrospray of curcumin-loaded microparticles for sustained drug release. PLoS One.

[16] Lee J., Sah H. (2022). Preparation of PLGA nanoparticles by milling spongelike PLGA microspheres. Pharmaceutics.

[17] Martins C., Sousa F., Araujo F. (2018). Functionalizing PLGA and PLGA derivatives for drug delivery and tissue regeneration applications. Adv. Healthc. Mater..

[18] Chen M., Shao Z., Chen X. (2012). Paclitaxel-loaded silk fibroin nanospheres. J. Biomed. Mater. Res..

[19] Zeng W., Hui H., Liu Z. (2021). TPP ionically cross-linked chitosan/PLGA microspheres for the delivery of NGF for peripheral nerve system repair. Carbohydrate. Polym..

[20] Dwivedi P., Yuan S., Han S. (2018). Core-shell microencapsulation of curcumin in PLGA microparticles: programmed for application in ovarian cancer therapy. Artif. Cells, Nanomed. Biotechnol..

[21] Shams T., Illangakoon U.E., Parhizkar M. (2018). Electrosprayed microparticles for intestinal delivery of prednisolone. J R Soc Interface.

[22] Vázquez-González Y., Prieto C., Filizoglu M.F. (2021). Electrosprayed cashew gum microparticles for the encapsulation of highly sensitive bioactive materials. Carbohydrate Polym..

[23] Han S., Dwivedi P., Mangrio F.A. (2019). Sustained release paclitaxel-loaded core-shell-structured solid lipid microparticles for intraperitoneal chemotherapy of ovarian cancer. Artif. Cells, Nanomed. Biotechnol..

[24] Wu D., Yu Y., Zhao C. (2019). NK-Cell-Encapsulated porous microspheres via microfluidic electrospray for tumor immunotherapy. ACS Appl. Mater. Interfaces.

[25] Zhang H., Xu R., Yin Z. (2022). Drug-loaded chondroitin sulfate microspheres generated from microfluidic electrospray for wound healing. Macromol. Res..

[26] Kevadiya B.D., Chettiar S.S., Rajkumar S. (2014). Evaluation of Montmorillonite/Poly (L-Lactide) microcomposite spheres as ambidextrous reservoirs for controlled release of Capecitabine (Xeloda) and assessment of cell cytotoxic and oxidative stress markers. Compos. Sci. Technol..

[27] Ding Y., Li W., Zhang F. (2019). Electrospun fibrous architectures for drug delivery, tissue engineering and cancer therapy. Adv. Funct. Mater..

[28] Yuan Y., Xu X., Gong J. (2019). Fabrication of chitosan-coated konjac glucomannan/sodium alginate/graphene oxide microspheres with enhanced colon-targeted delivery. Int. J. Biol. Macromol..

[29] Burmistrov I., Gorshkov N., Kovyneva N. (2020). High seebeck coefficient thermo-electrochemical cell using nickel hollow microspheres electrodes. Renew. Energy.

[30] Ren J., Chen L., Liu Y. (2021). Hollow cobalt phosphate microspheres for sustainable electrochemical ammonia production through rechargeable Zn–N_2_ batteries. J. Mater. Chem..

[31] Dwivedi P., Han S., Mangrio F. (2019). Engineered multifunctional biodegradable hybrid microparticles for paclitaxel delivery in cancer therapy. Mater. Sci. Eng. C.

[32] Dwivedi P., Kiran S., Han S. (2020). Magnetic targeting and ultrasound activation of liposome–microbubble conjugate for enhanced delivery of anticancer therapies. ACS Appl. Mater. Interfaces.

[33] Ni G., Yang G., He Y. (2020). Uniformly sized hollow microspheres loaded with polydopamine nanoparticles and doxorubicin for local chemo-photothermal combination therapy. Chem. Eng. J..

[34] Prajapati V.D., Jani G.K., Kapadia J.R. (2015). Current knowledge on biodegradable microspheres in drug delivery. Expet Opin. Drug Deliv..

[35] Zeng Z., Jiang G., Liu T. (2021). Fabrication of gelatin methacryloyl hydrogel microneedles for transdermal delivery of metformin in diabetic rats. Bio-Design. Manufact..

[36] Luo Z., Sun W., Fang J. (2019). Biodegradable gelatin methacryloyl microneedles for transdermal drug delivery. Adv. Healthc. Mater..

[37] Zhou X., Hou C., Chang T. (2020). Controlled released of drug from doubled-walled PVA hydrogel/PCL microspheres prepared by single needle electrospraying method. Coll. Surf. B: Biointer..

[38] Karamzadeh Y., Ansari Asl A., Rahmani S. (2020). PCL microsphere/PEG-based composite hydrogels for sustained release of methadone hydrochloride. J. Appl. Polym. Sci..

[39] Zhang Y., Wang Y., Wang B. (2022). Exclusive formation of poly(lactide) stereocomplexes with enhanced melt stability via regenerated cellulose assisted Pickering emulsion approach. Compos. Commun..

[40] Zhuang W., Ye G., Wu J. (2022). A 3D-printed bioactive polycaprolactone scaffold assembled with core/shell microspheres as a sustained BMP2-releasing system for bone repair. Biomater. Adv..

[41] Tu Y., Ren L., Shao J. (2022). Simultaneous removal of aniline and antimony (Sb(V)) from textile wastewater using amidoxime-PAN/PLA nanofiber microsphere supported TiO2. Sep. Purif. Technol..

[42] Liu Y., Yang L., Chen G. (2021). PBAT hollow porous microfibers prepared via electrospinning and their functionalization for potential peptide release. Mater. Des..

[43] Botta L., Titone V., Teresi R. (2022). Biocomposite PBAT/lignin blown films with enhanced photo-stability. Int. J. Biol. Macromol..

[44] Yan D., Wang Z., Guo Z. (2020). Study on the properties of PLA/PBAT composite modified by nanohydroxyapatite. J. Mater. Res. Technol..

[45] Li C., Cui Q., Li Y. (2022). Effect of LDPE and biodegradable PBAT primary microplastics on bacterial community after four months of soil incubation. J. Hazard Mater..

[46] Liu B., Guan T., Wu G. (2022). Biodegradation behavior of degradable mulch with poly (butylene adipate-co-terephthalate) (PBAT) and poly (butylene succinate) (PBS) in simulation marine environment. Polymers.

[47] Deshoulles Q., Gall M.L., Benali S. (2022). Hydrolytic degradation of biodegradable poly (butylene adipate-co-terephthalate) (PBAT) - towards an understanding of microplastics fragmentation. Polym. Degrad. Stabil..

[48] Ansary R., Rahman M., Mohamad N. (2017). Controlled release of lysozyme from double-walled poly (lactide-Co-glycolide) (PLGA) microspheres. Polymers.

[49] Lee W.L., Seh Y.C., Widjaja E. (2012). Fabrication and drug release study of double-layered microparticles of various sizes. J. Pharm. Sci..

[50] Lee W.L., Loei C., Widjaja E. (2011). Altering the drug release profiles of double-layered ternary-phase microparticles. J. Contr. Release.

[51] Zhu C., Wang J., Huang J. (2019). Preparation and evaluation of gastro-floating hollow adhesive microspheres of carbomer/ethyl cellulose encapsulating dipyridamole. New J. Chem..

[52] Li Y., Li Z., Zheng F. (2015). Polyaniline hollow microspheres synthesized via self-assembly method in a polymer acid aqueous solution. Mater. Lett..

[53] Wang S., Shi X., Gan Z. (2015). Preparation of PLGA microspheres with different porous morphologies. Chin. J. Polym. Sci..

[54] Sung H., Ferlay J., Siegel R.L. (2021). Global cancer statistics 2020: GLOBOCAN estimates of incidence and mortality worldwide for 36 cancers in 185 countries. CA: CA Cancer J. Clin..

[55] Siegel R.L., Miller K.D., Fuchs H.E. (2022). Cancer statistics, 2022. CA: CA Cancer J. Clin..

[56] Boobalana R., Kuang-Kai L., Jui-I C (2017). Synthesis and biological assay of erlotinib analogues and BSA-conjugated erlotinib analogue. Bioorg. Med. Chem. Lett..

[57] Abdelgalila A.A., Al-Kahtanib H.M., Al-Jenoobi F.I. (2019). Chapter four - erlotinib. Prof. Drug. Substan. Excipients. Relat. Methodol..

[58] Liu Y., Yang L., Chen G. (2021). PBAT hollow porous microfibers prepared via electrospinning and their functionalization for potential peptide release. Mater. Des..

[59] Bock N., Dargaville T.R., Woodruff M.A. (2012). Electrospraying of polymers with therapeutic molecules: state of the art. Prog. Polym. Sci..

[60] Wang X., Ju X., Sun S. (2015). Monodisperse erythrocyte-sized and acid-soluble chitosan microspheres prepared via electrospraying. RSC Adv..

[61] Mandal B., Balabathula P., Mittal N. (2012). Development and validation of a spectrofluorimetric method for the determination of erlotinib in spiked human plasma. J. Fluoresc..

